# Identification, RNAi Knockdown, and Functional Analysis of an Ejaculate Protein that Mediates a Postmating, Prezygotic Phenotype in a Cricket

**DOI:** 10.1371/journal.pone.0007537

**Published:** 2009-10-23

**Authors:** Jeremy L. Marshall, Diana L. Huestis, Yasuaki Hiromasa, Shanda Wheeler, Cris Oppert, Susan A. Marshall, John M. Tomich, Brenda Oppert

**Affiliations:** 1 Department of Entomology, Kansas State University, Manhattan, Kansas, United States of America; 2 Biotechnology Core Laboratory, Kansas State University, Burt Hall, Manhattan, Kansas, United States of America; 3 Department of Entomology and Plant Pathology, University of Tennessee, Knoxville, Tennessee, United States of America; 4 Department of Biochemistry, Kansas State University, Manhattan, Kansas, United States of America; 5 USDA ARS Grain Marketing and Production Research Center, Manhattan, Kansas, United States of America; University of Texas Arlington, United States of America

## Abstract

Postmating, prezygotic phenotypes, especially those that underlie reproductive isolation between closely related species, have been a central focus of evolutionary biologists over the past two decades. Such phenotypes are thought to evolve rapidly and be nearly ubiquitous among sexually reproducing eukaryotes where females mate with multiple partners. Because these phenotypes represent interplay between the male ejaculate and female reproductive tract, they are fertile ground for reproductive senescence – as ejaculate composition and female physiology typically change over an individual's life span. Although these phenotypes and their resulting dynamics are important, we have little understanding of the proteins that mediate these phenotypes, particularly for species groups where postmating, prezygotic traits are the primary mechanism of reproductive isolation. Here, we utilize proteomics, RNAi, mating experiments, and the *Allonemobius socius* complex of crickets, whose members are primarily isolated from one another by postmating, prezygotic phenotypes (including the ability of a male to induce a female to lay eggs), to demonstrate that one of the most abundant ejaculate proteins (a male accessory gland-biased protein similar to a trypsin-like serine protease) decreases in abundance over a male's reproductive lifetime and mediates the induction of egg-laying in females. These findings represent one of the first studies to identify a protein that plays a role in mediating both a postmating, prezygotic isolation pathway and reproductive senescence.

## Introduction

Answers to many of evolutionary biology's biggest questions lie in understanding the production and interactions of sex-specific, reproductive tract proteins. Insights into questions such as ‘what determines successful fertilization?’, ‘what genes underlie sperm competition and sexual conflict?’, ‘what mechanisms influence the evolution of postmating, prezygotic isolation?’, and ‘why do reproductive tract genes evolve more rapidly than non-reproductive genes?’ depend on an understanding of the functions, interactions, and evolution of reproductive tract proteins [e.g.], [ 1–2]. In species with internal fertilization, a particularly important group of reproductive proteins are those that are transferred from the male to the female during copulation – i.e., ejaculate proteins. The importance of ejaculate proteins is easy to understand, as sperm and seminal fluid proteins not only mediate successful sperm-egg interactions [e.g.], [3]–[8] but often regulate physiological processes such as sperm storage [9]–[11], a male's probability of paternity [12]–[13], induction of egg-laying [13]–[14], female attractiveness [15], and even life span [16].

The advent of genetic tools such as RNAi [17] has enabled researchers to identify the genetic mechanisms underlying a range of physiological traits, including egg-production and sexual receptivity in *Drosophila* [e.g., 14]. Studies on insect systems especially have benefited from RNAi technology; indeed, injection of dsRNA or siRNA into adult or juvenile insects has been a successful strategy to knockdown gene transcripts in a diverse array of taxa, including aphids [e.g., *Acyrthosiphon pisum*; 18], beetles [*Tribolium castaneum*; 19], cockroaches [e.g., *Blattella germanica*; 20], field crickets [e.g., *Gryllus bimaculatus*; 21], fruit flies [e.g., *Drosophila melanogastor*; 22], grasshoppers [e.g., *Schistocerca americana*; 23], honeybees [e.g., *Apis mellifera*; 24], moths [e.g., *Spodoptera frugiperda*; 21], mosquitoes[e.g., *Anopheles gambiae*; 25], and termites [e.g., *Reticulitermes flavipes*; 26]. Thus, RNAi technology, in combination with studies on ejaculate and reproductive tract proteins in insects, offers an opportunity to assess the function of individual proteins and their role in mediating reproductive physiologies.

**Figure 1:**
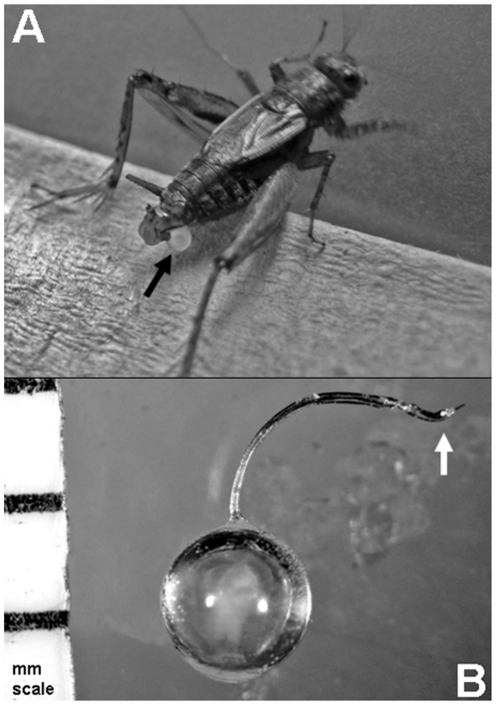
The spermatophore. The spermatophore is the sperm- and accessory gland fluid-filled package that male crickets produce and hold externally (A) prior to copulation. During copulation, the male transfers the spermatophore (B) to the female via threading the tip of the spermatophore duct (see arrow in B) into the reproductive tract of the female. The white material inside the spermatophore in (B) is the ejaculate.

Unfortunately, it is often difficult to study ejaculate proteins in non-vertebrate systems with internal fertilization. The primary reason for this difficulty is the all-too-often inability to collect whole ejaculates just prior to insemination (i.e., an ejaculate that does not suffer from being incomplete because it is still inside the male or being “contaminated” by female proteins just after insemination). However, there are insect species, like the ground cricket *Allonemobius socius*, where males produce an external ejaculate (Fig. 1A) that is surrounded by a protective protein coat which not only encapsulates the ejaculate, but allows for internal insemination during copulation via a small duct (see arrow in Fig. 1B). This entire structure, including ejaculate, is called a spermatophore (Fig. 1B) and provides a means by which researchers can study the contents and variation in ejaculates just prior to insemination. With this biological feature in hand, ejaculate proteins can be studied both independently and in the context of protein-protein interactions within the female's reproductive tract.

**Figure 2:**
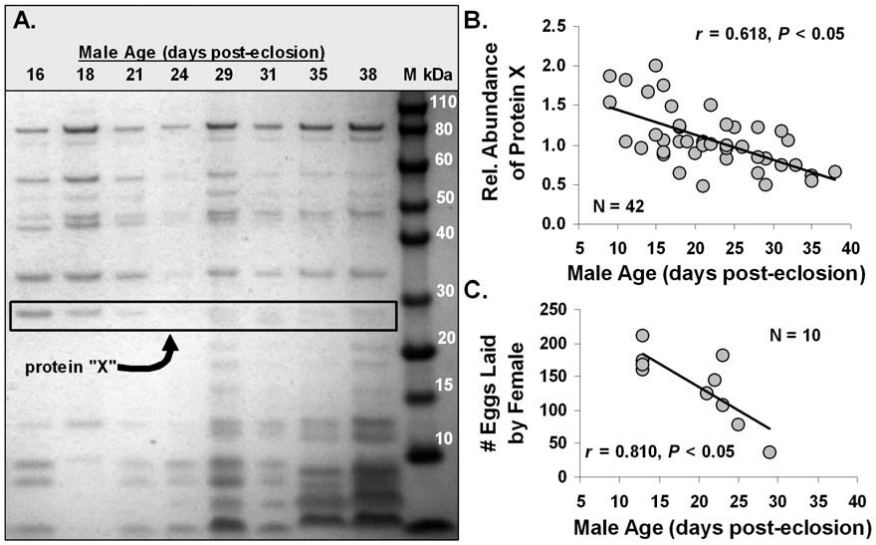
Protein “X” and the effects of male age. The decrease in abundance of protein “X” in a male's ejaculate is associated with increased male age (A; each lane represents the protein from a single spermatophore), while (B) demonstrates the statistical significance of this relationship (each point represents the data from a single spermatophore). (C) The association between male age and a male's ability to induce a female to lay eggs – suggesting that the relative abundance of protein “X” may be linked to a male's ability to induce a female to lay eggs.

The *A. socius* complex of crickets (including the species *A. socius*, *A. fasciatus*, and *A. sp. nov.* Tex) has been a model system within ecology and evolutionary biology for nearly three decades and has been at the forefront of studies assessing the importance of postmating, prezygotic reproductive isolation [27]–[32]. Indeed, research has shown that postmating, prezygotic phenotypes, such as conspecific sperm precedence [CSP; 28]–[30] and the ability of a male to induce a female to lay eggs [27], [30], [31], isolate species in this complex, while phenotypes such as calling song [33], mating/courtship behavior [34], phenology [35]–[37], and postzygotic phenotypes [27] do not. Additionally, research on the effects of male age on ejaculate composition and postmating, prezygotic phenotypes has uncovered several patterns. Specifically, one of the most abundant proteins in the ejaculate, initially called protein “X”, decreases with male age (Fig. 2A, B). Older males are also less able to induce females to lay eggs (Fig. 2C) – a form of reproductive senescence. Together, these data suggest the hypothesis that the abundance of protein “X” underlies a male's ability to induce a female to lay eggs. If confirmed, this protein would not only be linked to male reproductive senescence, but also as a critical player in one of the postmating, prezygotic phenotypes that isolate species in this complex of crickets.

Here, using the *A. socius* complex of crickets, our goals were to identify protein “X” using biochemical and genetic analyses, sequence and clone the full length transcript that produces protein “X”, assess tissue- and sex-specificity of this transcript, and use RNAi technology to knockdown transcript expression and evaluate the phenotypic effects. We found that protein “X” is a male accessory gland-biased protein exhibiting the molecular features of a trypsin-like serine protease. Additionally, we provide evidence that this protein mediates a male's ability to induce a female to lay eggs – which is a phenotype that contributes to both male reproductive senescence and postmating, prezygotic reproductive isolation.

## Results

### Identification of an abundant ejaculate protein

Protein “X” (Fig. 2), an approximately 29 kDa protein, was excised from a 1D-SDS-PAGE gel, trypsin digested, and analyzed via MALDI-TOF and MALDI-TOF/TOF-MS analyses. Seven high intensity MS peaks of peptides were then subjected to MS/MS analysis, with the resulting peptide sequences searched against the male reproductive accessory gland EST database from *A. fasciatus* (GenBank accession numbers: EG018565-EG019055) using MASCOT (http://www.matrixscience.com). Four of the seven peptides (Table 1) corresponded to a single transcript derived from a contig of seven *A. fasciatus* ESTs (GenBank accession numbers: EG018587, EG018591, EG018599, EG018669, EG018803, EG018819, EG018935). The amino acid sequences of these four peptides exhibited 100% sequence identity to peptides from this EST contig and had significant MASCOT MS/MS scores (Table 1).

**Table 1:** Peptides generated with MS/MS analyses and their percent match to an EST contig from the male reproductive accessory gland.

Peptide #	Peptide Sequence	No. ofamino acids	% Match toESTcontig	MASCOTMS/MS score	Accession #scontaining this peptide
1	KEDLTVVLGLHDR	13	100%	58	EG018587, EG018935, EG018803
2	GQDIYADQVAFVTGWGR	17	100%	150	EG018599, EG018669, EG018819
3	VEQIGVVSWGIGCAR	15	100%	70	EG018591, EG018669, EG018819
4	PGMPGVYTTVSYYLDWIR	18	100%	20	EG018591, EG018669

Note: Individual ion scores from the MS/MS analysis that are >56 indicate significant homology (*P*<0.05).

**Figure 3:**
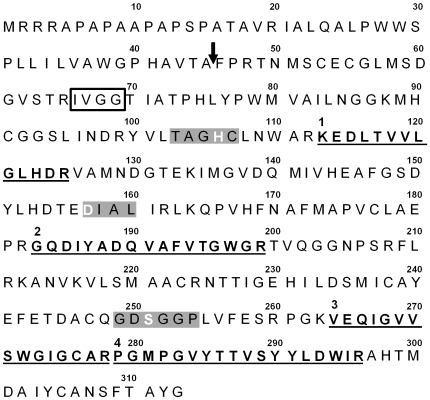
Amino acid sequence of protein “X”. The features of the protein “X” sequence include a predicted signal peptide (the sequence 5′ of the arrow) and cleavage site (at the arrow), a trypsin-like N-terminal motif (open box around IVGG), the three catalytic residues (white letters) in the three conserved motifs (gray boxes) of a serine protease, and the four peptides recovered from MS/MS analysis (labeled 1–4 and highlighted in bold and underlined).

From this male accessory gland EST contig, we developed primers to amplify the entire coding region of this transcript (see Methods below). Following PCR amplification of this transcript from male accessory gland cDNA, we cloned and sequenced the resulting product (NCBI accession #GQ911573). The gene nucleotide sequence from these clones was identical to that of the EST contig and was translated to a 313 amino-acid residue sequence [Fig. 3; the four significant peptide matches are denoted 1 through 4 (as in Table 1) with the sequences being in bold and underlined]. The translated sequence contains a signal peptide, presumably cleaved between Ala^45^ and Phe^46^ (SignalP 3.0 probability  = 0.957), resulting in a mature protein of 268 amino-acid residues (Fig. 3). The calculated molecular mass of this protein, 29.3 kDa, was in agreement with the estimated mass based on migration in the 1D SDS-PAGE gel (Fig. 2A).

A BLASTP search identified a trypsin-like serine protease motif between residues Ile^66^ and Met^300^, beginning with the conserved N-terminal motif IVGG (Fig. 3; box around IVGG motif). This protein contains the catalytic triad His^106^, Asp^157^, and Ser^251^ characteristic of serine proteases in the S1 family (numbering based on the uncleaved protein; Fig. 3). These catalytic residues were found in the highly conserved motifs TAGHC, DIAL, and GDSGGP, respectively (Fig. 3; [38]). Based on the above features, we propose that this protein is a functional trypsin-like serine protease.

Given that this transcript appears to be a trypsin-like serine protease and such proteases are common in many tissues, we evaluated the tissue-and sex-specificity of this and other trypsin-like serine proteases. To accomplish this, we developed conserved primers in the IVGG and DIAL amino acid regions (see Methods below). These primers amplified trypsin-like serine proteases from the following tissues: male accessory gland, male testis, male thorax, male digestive tract, female spermatheca, female ovaries, female thorax, and female digestive tract. Next, we conducted 5′RACE using primers in the IVGG and DIAL regions as the inner and outer primers respectively (see Methods below), and found considerable variation between different tissues and males and females (Table 2). Specifically, from a total of 137 sequenced clones, we identified 19 unique trypsin-like serine protease sequences, and significantly, the male accessory gland yielded a single transcript that was not found in the other male tissues nor in any tissue from females (sequence #5, Table 2). This particular transcript also has not been found in our testis and female reproductive tract EST libraries, which consist of >30,000 EST generated with 454 sequencing (unpubl. data). Although this particular trypsin-like serine protease appears unique to the male accessory gland, it is still possible that it is a rare transcript in these other tissues and within females. Therefore, we suggest that this transcript is at least male- and accessory gland-biased and potentially uniquely expressed in this tissue.

**Table 2:** BLASTP analysis of the full length transcript underlying protein “X”.

Scientific Name	Common Name	GenBank accession #	E-value	Gene Identity
*Gryllus firmus*	sand field cricket	ABG75840	1e-142	hypotheical Acp
*Gryllus pennsylvanicus*	fall field cricket	ACD69515	8e-117	trypsin-like serine protease
*Nasonia vitripennis*	jewel wasp	XP_001606267	8e-76	hypotheical protein
*Bombyx mori*	domestic silkworm	NP_001153675	8e-76	male reproductive organ serine protease 2
*Tribolium castaneum*	red flour beetle	XP_974113	5e-50	similar to oviductin
*Drosophila melanogaster*	fruit fly	NP652645	2e-46	CG18735

As for amino-acid sequence similarity to proteins from other organisms, our BLASTP search found a nearly identical transcript in the field cricket (*Gryllus firmus*) EST library from the male reproductive accessory gland (1e^−144^; Table 3). In *G. firmus*, this appears to be a male-biased transcript (e.g., AG-0308F; Fig. 2 in ref. [39]) that, like in *Allonemobius*, is produced in the male reproductive accessory gland. This *A. socius* transcript also is similar to S1 family serine proteases in other insects (Table 3). Given the male-biased expression of this transcript in the accessory gland and its presence in the male ejaculate, we name the gene encoding this transcript *ejaculate serine protease* – with the protein and gene being abbreviated EJAC-SP and *ejac-sp*, respectively.

**Table 3:** The occurrence of specific sequences encoding putative trypsin-like serine protease in various male and female cricket tissues.

		Male Tissues								Female Tissues							
		ACG		Testes		Thorax		Dig. Track		Spmthca.		Ovaries		Thorax		Dig. Track	
#	Sequence (20 bp 5′ of IVGG)	N	(%)	N	(%)	N	(%)	N	(%)	N	(%)	N	(%)	N	(%)	N	(%)
1	GAAGACATCGCCCTCATCCG					5	38.5	1	6.7	9	40.9	3	18.8	1	6.3		
2	TGTATTCCCCGATGTGTCTT							2	13.3								
3	TAATGCTGGCGGCTGAATCG							2	13.3								
4	CGAGATAATGATTAATGGGG			3	30.0	7	53.8	10	66.7	7	31.8						
5	CTGACGGTGTCTCAACACGT	29	100.0														
6	CCCTCAGCCCGTTGACCGGT					1	7.7										
7	AGAAGAATAGGGTGGGTGAC			3	30.0												
8	CCTAAGATTTTATTTTGAAC			4	40.0												
9	ACATTGAGGGAAAATTGAGA													15	93.7		
10	CCGGCAGCCTCAGCAGCCGC															16	100.0
11	GGTCTGTACGATTTGGTTTC											5	31.3				
12	CGTTTGCTGGCTTTGATGAA											1	6.3				
13	ACGAGGGGAAGAGGAGTATA											1	6.3				
14	TATCTTCAACGTAAGCTGAT											4	25.0				
15	TAATTGATTATATTGAAGAC											1	6.3				
16	CAGGACGTGGACCAACCCAC											1	6.3				
17	GTTCATTCATCGCCATCAGC									1	4.5						
18	GCGCGCGTGCGGCCCAGAAC									1	4.5						
19	CCCGAGCCGTCTTCCCCTCC									4	18.3						

ACG  =  reproductive accessory gland; Dig. Tract  =  digestive tract; Spmthca.  =  spermatheca.

### RNAi knockdown and functional analysis of *ejac-sp*

**Figure 4:**
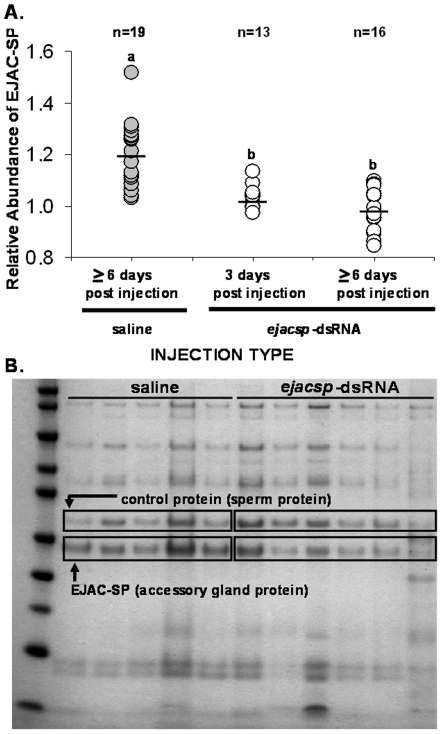
RNAi and the knockdown of EJAC-SP protein in the male ejaculate. The relative abundance of EJAC-SP protein in saline- and *ejacsp*-dsRNA-injected males at three and/or six days post-injection (A; a and b refer to statistically different groups based on post-hoc tests, *P*<0.00001). The gel (B) is a representative 1D-SDS PAGE gel of proteins from individual spermatophores from males in both the saline- and *ejacsp*-dsRNA treatments at ≥6 days post-injection. The EJAC-SP protein and the protein used as a control are outlined in boxes.

We developed *ejac-sp* specific primers to amplify a 99 bp region that is 5′ to the conserved IVGG site (Fig. 3) and unique to this transcript (see Methods below). We made *ejacsp*-dsRNA, following standard protocols, and injected it into the abdomen of adult male crickets (see below). We found that males injected with *ejacsp*-dsRNA produced spermatophores with significantly reduced levels of the EJAC-SP protein in their ejaculates, relative to the saline controls (*F*_2,45_ = 22.1, *P*<0.00001; Fig. 4A&B). Significant knockdown was seen 3 days post-injection and was still evident 6 days post-injection (additional experiments, data not shown, suggest that RNAi knockdown is effective for 12 days post-injection and can last the entire reproductive life of the male). At 6 days post-injection, ∼79% (15 of 19 individuals) of saline-injected males produced spermatophores with an EJAC-SP relative abundance of 1.1, while none (0 of 16 individuals) of the *ejacsp*-dsRNA injected males produced spermatophores with relative abundances this high (Fig. 4A). Conversely, ∼56% (9 of 16 males) of *ejacsp*-dsRNA injected males produced spermatophores with EJAC-SP relative abundances of less than one, while none of the saline-injected males had abundances this low (0 of 19 males; Fig. 4A).

**Figure 5:**
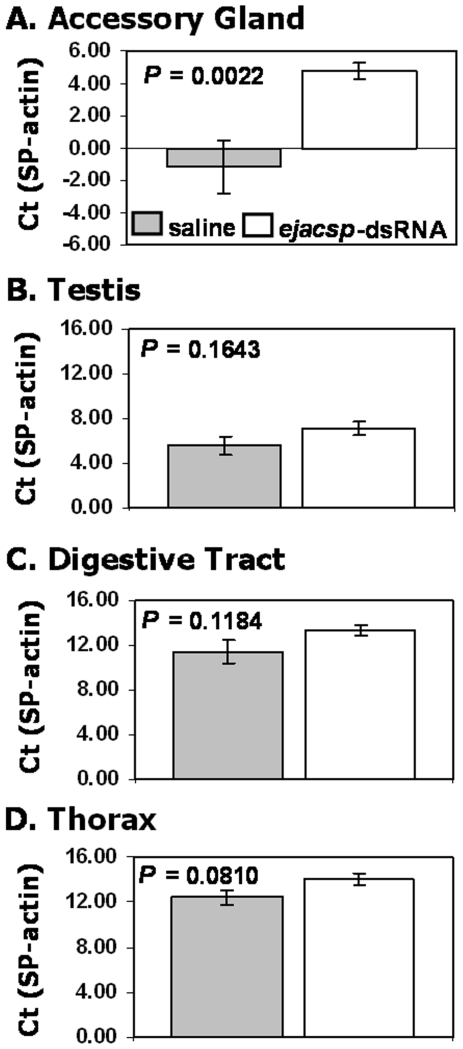
Real-time PCR of trypsin-like serine protease in each tissue for each injection treatment. The expression, as quantified by ΔCt via qPCR, of trypsin-like serine protease relative to *β-actin* (the control gene) in the saline- and *ejacsp*-dsRNA-injected males (gray and open bars, respectively) for the male reproductive accessory gland (A), testis (B), digestive tract (C), and thorax (D). Significant knockdown only occurred in the male accessory gland. Sample size equals 11 in both treatments for all tissue types.

The above data suggest that our RNAi approach was successful in knocking down the abundance of EJAC-SP protein in the male ejaculate. To evaluate knockdown in the male accessory gland, testes, digestive tract, and thorax, we conducted quantitative real-time PCR (qPCR) using conserved trypsin-like serine protease primers for the target gene and *actin* primers for the housekeeping gene (see Methods below). We found significant knockdown of trypsin-like serine proteases in the male accessory gland of *ejacsp*-dsRNA injected males (*t* = 3.52, *P* = 0.0022, n = 11 per treatment for all tissues; Fig. 5A). This significant difference translated into a 63-fold average difference between the *ejacsp*-dsRNA and saline injected treatments. However, there were no significant differences between the *ejacsp*-dsRNA- and saline-injected treatments for the remaining three tissues (Fig. 5B–D; testis: *t* = 1.44, *P* = 0.1643; digestive tract: *t* = 1.63, *P* = 0.1184; thorax: *t* = 1.84, *P* = 0.0810).

**Figure 6:**
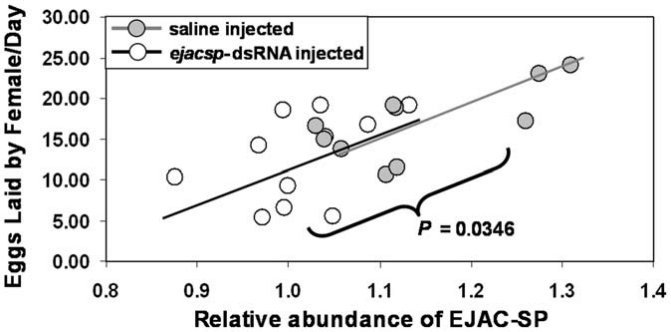
Phenotypic effects of *ejac-sp* knockdown. The abundance of EJAC-SP protein in a spermatophore (taken from a male just prior to being allowed to copulate successfully with a virgin female) predicts a male's ability to induce a female to lay eggs. The difference between saline- and *ejacsp*-dsRNA injected males was also significant (*P* = 0.0346).

The phenotypic effect of knocking down *ejac-sp* expression, yielding reduced amounts of EJAC-SP protein in the male ejaculate, was a significantly reduced ability to induce a female to lay eggs [number of eggs laid per day: μ_saline_ = 16.93 (n = 10), μ*_ejacsp_*_-dsRNA_ = 12.73 (n = 11); *t*_μ1>μ2_ = 1.925, *P* = 0.0346; Fig. 6]. This knockdown represented an approximately 25% reduction in egg-laying rates relative to the saline control. Overall, there was a significant, linear relationship between the relative abundance of EJAC-SP in the male ejaculate and the number of eggs laid per day by females (*F*_1,19_ = 13.06, *P* = 0.0018; *r* = 0.638; Fig. 6). These results are consistent with the hypothesis that the abundance of protein “X” (identified here as EJAC-SP) mediates a male's ability to induce a female to lay eggs.

## Discussion

Our primary goals for conducting this research were to identify protein “X”, which we now refer to as EJAC-SP (a product of the gene *ejaculate serine protease*), and determine if the correlation between decreased levels of EJAC-SP in the male ejaculate and decreased ability of males to induce females to lay eggs are causally linked or each independently related to male age (Fig. 2). With regard to protein identity, we found that EJAC-SP has molecular characteristics of a trypsin-like serine protease and that the *ejac-sp* transcript exhibits a male, reproductive accessory gland-biased pattern of expression (Table 3). Indeed, *ejac-sp* appears to be exclusively expressed in the accessory gland of males, but further research is needed to distinguish between “biased”- and “exclusive”- expression. Such sex-biased expression is not uncommon, especially for reproductive tract genes [e.g.], [40]–[42].

To address the question of *ejac-sp* function, we evaluated the utility of a common RNAi protocol. We found that injecting gene-specific dsRNA (i.e., *ejacsp*-dsRNA) into the abdomen of adult male crickets resulted in a significant, 63-fold decrease in transcript abundance of trypsin-like serine proteases in the male accessory gland, but no significant knockdown in other male tissues (Fig. 5). This significant knockdown of transcript expression resulted in a significant decrease of EJAC-SP protein levels in the male ejaculate (Fig. 4) – even though all males in the saline and *ejacsp*-dsRNA treatments were between 14 and 22 days post-eclosion (i.e., reproductively young males given sexual maturity is usually reached 10 to 14 days post-eclosion). In terms of RNAi effectiveness, at least 56% of dsRNA-injected males exhibited significant knockdown of EJAC-SP, as these males had EJAC-SP relative abundances lower than any saline-injected male. Together, these results suggest that injecting gene-specific dsRNA into the abdomen of adult *Allonemobius* crickets can knockdown the abundance of a targeted transcript within a specific tissue, indicting the occurrence of a systemic RNAi pathway. Once again, this is not unexpected given that a systemic RNAi response has been found in other Orthopterans [e.g., crickets [21] and grasshoppers [23]] and basal insects [e.g., cockroaches [20] and termites [26]].

As for phenotype, we found a significant, linear relationship between EJAC-SP abundance in a male's ejaculate and a male's ability to induce a female to lay eggs (Fig. 6). This relationship parallels the hypothesized relationship derived from naturally-occurring variation linked to male age (Fig. 2). Moreover, RNAi knockdown of *ejac-sp* expression, and the resulting reduction in EJAC-SP protein, yields the predicted decrease in a male's ability to induce a female to lay eggs (Fig. 6). These results support a causal link between EJAC-SP abundance in the male ejaculate and a male's ability to induce a female to lay eggs.

The occurrence of “egg-laying induction” proteins in the ejaculate of male insects is expected, as several have been identified in *Drosophila* [reviewed in 43]. The fact that a serine protease was identified as an important part of the male's egg-laying induction pathway is also consistent with previous research, as serine proteases and serine protease inhibitors play critical roles in many postmating, prezygotic phenotypes [reviewed in 44]. Although more work is needed to identify the function and biochemical target of EJAC-SP, we hypothesize that its function is to cleave a specific protein in the female reproductive tract that initiates the egg-laying pathway; we are currently working to assess the function and biochemical target of EJAC-SP.

The natural decrease in EJAC-SP abundance as males get older not only helped identify this protein as an egg-laying induction candidate, but also represents a form of reproductive senescence. Such senescence is widespread among animals and typically results in decreased fitness of older males [e.g.], [45]–[47]. The form of reproductive senescence seen in *Allonemobius*, that of a changing in the quality or size of ejaculate as males age, can be found in animals ranging from insects [e.g.], [48], [49] to mammals [e.g.], [50], [51] and would appear to be a common form of male reproductive senescence. Unfortunately, except for the literature on the genetic basis of reproductive senescence in human males [e.g., 51], relatively few genes and proteins that underlie male reproductive senescence have been identified and functionally evaluated. Therefore, to our knowledge, EJAC-SP is one of the first non-mammalian proteins to be directly linked with male reproductive senescence.

The link between EJAC-SP abundance to a male's ability to induce a female to lay eggs provides the first insight into the genes, and resulting proteins, that mediate one of the main postmating, prezygotic phenotypes that reproductively isolate species in the *Allonemobius socius* complex of crickets. The proposed serine protease activity of EJAC-SP may or may not contribute to the species-specific ability of conspecific males to induce females to lay eggs – although we are currently evaluating this possibility. As stated above, we are in the process of identifying the function and target of EJAC-SP and once identified, we will be in a position to evaluate the degree to which species-specificity of this postmating, prezygotic phenotype is controlled by structural variation in the EJAC-SP protein. However, regardless the role played by EJAC-SP in reproductively isolating species, it does play a role in this postmating, prezygotic phenotype and is a part of the molecular pathway that underlies a component of reproductive isolation – a pathway that through the “magic” of biochemistry we will be able to identify the species-specific components.

In conclusion, the above results indicate that RNAi can be a useful technique to manipulate the ejaculate proteins of adult male crickets. This tool enables researchers to assess the function and importance of individual ejaculate proteins in determining fertilization success, the outcome of sperm competition, and the genes that underlie postmating, prezygotic isolation. Moreover, given that non-genetic model insects, particularly crickets, butterflies, and water striders [reviewed in 52], [53], are key model systems for addressing questions about sexual selection and sexual conflict, the success of this technique demonstrates the power of combining proteomic/biochemical techniques and RNAi technology to address these types of questions in systems that have been studied for decades, yet currently lack a sequenced genome.

## Materials and Methods

### Spermatophores: sample preparation, SDS-PAGE, and estimates of abundance

Spermatophores were collected from males (both *A. socius* and *A. sp. nov.* Tex were used for all analyses) non-destructively, thus allowing multiple spermatophores to be collected from individual males throughout their reproductive lives. Specifically, a single male was paired with a single female and allowed to proceed through the normal courtship ritual [outlined in 54]. During this courtship ritual, males produce a spermatophore and hold it externally with their genitalia (Fig. 1). Once produced, it takes approximately 12 minutes for the outer protein coat of the spermatophore to harden and become structurally sufficient for successful ejaculate transfer. Following this time period, males will initiate copulation via a ritualized calling song and copulation dance. Just prior to copulation, we would disturb the courtship, anesthetize the male with CO_2_, remove the spermatophore, and store it at −80°C until used for protein analysis.

Prior to gel electrophoresis, spermatophores were ground and sonicated in 15–20 µL of purified water and then centrifuged for 30 sec at 12,000 rpm. The protein content of the resulting supernate was quantified with a ND-1000 (NanoDrop Industries, Wilmington, DE) spectrophotometer by A_280_. A typical spermatophore contained 30–80 µg of water-soluble protein.

For protein quantification, 25 µg of spermatophore proteins were separated on 4–12% Bis-Tris SDS-PAGE (using an Invitrogen NuPAGE system) following manufacturer's protocols (Fig. 2A). After staining with Coomassie Blue and de-staining with water, gel images were digitized with a Kodak Gel Logic 200. The amount of EJAC-SP (Fig. 4B) was compared to a control protein band, which was the 31 kDa protein band just above EJAC-SP (Fig. 4B, see box; which MALDI TOF/TOF MS indicates is sperm specific – data not shown). Protein bands were quantified by densitometry using ImageJ 1.37v software from NIH (rsbweb.nih.gov/ij/). Specifically, the amount of protein was estimated as maximum gray value for each protein band. The relative amount of EJAC-SP for each spermatophore was calculated as “EJAC-SP abundance/control protein abundance”.

### Protein identification with MALDI-TOF/TOF MS and MS/MS

After staining gels with Coomassie G-250, the selected gel band (protein “X” in Fig. 2A) was excised as 1–2 mm diameter pieces and transferred to a 1.5 mL Eppendorf tube. A protein-free region of the gel was also excised as background control. The control and test gel sections were destained using three 30 min washes of 60 µL 1∶1 acetonitrile: water at 30°C. Gel pieces were then dried for 10 min under vacuum. The gel sections were subjected to reduction and alkylation using 50 mM Tris (2-carboxyethyl) phosphine (TCEP) at 55°C for 10 min followed by 100 mM iodoacetamide in the dark at 30°C for 60 min. The carboxymethylated gels were thoroughly washed and re-dried in vacuo, then incubated with sequencing grade trypsin (Trypsin Gold, Promega, Madison, WI), 20 ng/µL in 40 mM ammonium bicarbonate, in 20 µL. Upon rehydration of the gels, an additional 15 µL of 40 mM ammonium bicarbonate and 10% acetonitrile was added, and gel sections were incubated at 30°C for 17 h in sealed Eppendorf tubes. The aqueous digestion solutions were transferred to 1.5 mL clean Eppendorf tubes, and tryptic fragments remaining within the gel sections were recovered by a single extraction with 50 µl of 50% acetonitrile and 2% trifluoracetic acid (TFA) at 30°C for 1 h. The acetonitrile fractions were combined with previous aqueous fractions and the liquid was removed by speed vacuum concentration. The dried samples were resuspended in 10 µL of 30 mg/mL 2,5-dihydroxylbenzonic acid (DHB) (Sigma, St. Louis, MO) dissolved in 33% acetonitrile/0.1% TFA and 2 µL of peptide/matrix solution was applied on a Bruker Massive Aluminum plate for MALDI-TOF and TOF/TOF analysis.

*MS and MS/MS analysis* - Mass spectra and tandem mass spectra were obtained on a Bluker Ultraflex II TOF/TOF mass spectrometer. Positively charged ions were analyzed in the reflector mode. MS and MS/MS spectra were analyzed with Flex analysis 3.0 and Bio Tools 3.0 software (Bruker Daltonics). Measurements were externally calibrated with 8 different peptides ranging from 757.39 to 3147.47 (Peptide Calibration Standard I, Bruker Daltonics) and internally re-calibrated with peptides from the autoproteolysis of trypsin. Peptide ion searches were performed with EST_others_20080308 in NCBInr database (as well as an EST database specific to these crickets) using MASCOT software (Matrix Science). The following parameters were used for the database search: MS and MS/MS accuracies were set to <0.6Da, trypsin/P as an enzyme, missed cleavages 1, carbamidomethylation of cysteine as fixed modification, and oxidation of methionine as a variable modification. Homology of the predicted protein sequence was searched in NCBI database with Blast 2.0.

### Cloning the full-length transcript of *ejac-sp*

Using a contig of *ejac-sp* ESTs derived from the male reproductive accessory gland EST library (GenBank accession numbers: EG018587, EG018591, EG018599, EG018669, EG018803, EG018819, EG018935), we developed primers to sequence the entire coding region (forward primer: Ovi-Full-F2, CGCTTCTGACAGCCATGC; reverse primer, Ovi-R-985a, CGCTACTCCTTATCCGTACCTTGCT). These primers were used with standard PCR reaction chemistry for a 50 µL reaction (outlined in 31), 100 ng of male accessory gland-specific first-strand cDNA [generated by isolating RNA with an Ambion RNAqueous-4PCR kit and standard protocols for 1^st^-strand cDNA synthesis (i.e., using 8 µL of the total RNA solution, 5 µL 5X RT buffer, 1.3 µL dNTP's, 0.7 µL rRNasin, 1 µL M-MLV reverse transcriptase, 2 µL of poly-T primer and nuclease-free water to 20 µL - all reagents from Promega, Madison, WI)], and a thermocycler profile of 94°C for 2 mins, 30 cycles of 94°C for 30 sec, 55°C for 30 sec, 72°C for 1 min, and a final extension period of 72°C for 7 min. The resulting PCR product was run on a 1% agarose gel and gel extracted using a Qiagen QIAquick Gel Extraction Kit. The cleaned PCR product was cloned using a TA Cloning® Kit (Invitrogen) and sequenced with standard M13 forward and reverse primers.

### Tissue- and sex-specificity of *ejac-sp* and other trypsin-like serine proteases

To determine the presence of trypsin-like serine protease transcripts in a variety of male and female cricket tissues (i.e., male accessory gland, male testis, male thorax, male digestive tract, female spermatheca, female ovaries, female thorax, and female digestive tract), we developed nucleotide primers in the conserved amino-acid motifs IVGG and DIAL (forward primer in IVGG region, ovi F 230con, ATCGTCGGGGGCACAATC; reverse primer in DIAL region, ovi R 500con, CGGATGAGGGCGATGTCTTC). These primers yield a ∼290 bp fragment that was present in all tissues sampled from both sexes. Next, we utilized the ovi R 500con primer (i.e., the reverse primer in the DIAL region) and the reverse complement of the ovi F 230con primer (i.e., ovi R 230con, GATTGTGCCCCCGACGAT, which is in the IVGG region) as the gene-specific outer and inner primers, respectively, for 5′RACE for each tissue within each sex. For 5′RACE, we utilized the FirstChoice® RLM-RACE kit from Ambion. Following 5′RACE on all eight samples, we cloned (using the TA Cloning Kit) and sequenced the resulting products. We sequenced 10 to 30 clones per sample with an average of 17 (i.e., 137 sequenced clones in total). The resulting sequences were analyzed for the occurrence of unique sequence 5′ to the conserved IVGG region.

### Preparation and injection of dsRNA

Following the identification of a unique region 5′ of the IVGG site for the *ejac-sp* transcript, we developed *ejac-sp* specific primers to amplify a 99 bp fragment in this unique region (forward primer, ovi F 140, TACTCATCTTGGTGGCCTG; reverse primer, Ovi R 209, GTGTTGAGACACCGTCAGACA). We added the T7 promoter sequence (TAATACGACTCACTATAGGGAGA) to the 5′ end of each primer. Our final 5′ primer was TAATACGACTCACTATAGGGAGA TACTCATCTTGGTGGCCTG and our final 3′ primer was TAATACGACTCACTATAGGGAGA GTGTTGAGACACCGTCAGACA (with the underlined sections being the T7 region). These primers were used with standard PCR reaction chemistry for a 50 µL reaction (same as above), 1 µg of male accessory gland-specific first-strand cDNA (isolated as above), and a thermocycler profile of 94°C for 2 min, 30 cycles of 94°C for 30 sec, 55°C for 30 sec, 72°C for 1 min, and a final extension period of 72°C for 7 min. The resulting PCR product was isolated from a 1% agarose gel using a Qiagen QIAquick Gel Extraction Kit. The cleaned PCR product was subjected to a standard ethanol precipitation to yield a final concentration of greater than 111 ng/µL, as measured by a ND-1000 spectrophotometer.

The Ambion T7 MEGAscript kit was used to generate *ejacsp*-dsRNA via RNA transcription. We followed the specified manufacturer's protocol, except that we increased reactions to 30 µL for a higher yield of dsRNA. We also used 1 µg of template DNA (i.e., our concentrated PCR product). The RNA transcription reaction was incubated at 37°C for 14–16 h. Following incubation, the reaction was subjected to DNase treatment following T7 MEGAscript kit guidelines. The dsRNA was then cleaned using Ambion's MEGAclear Kit, resulting in a ready-to-inject dsRNA solution. The concentration of dsRNA was checked with a ND-1000 spectrophotometer and the volume adjusted to a final concentration of 1 µg/µL.

For injections, we used a manual injection system consisting of a syringe and disposable glass needles. We injected adult males in the abdomen with 1 µL of either *ejacsp*-dsRNA or saline depending on the experimental treatment. To accomplish the injections, we anesthetized adult males with CO_2_ and performed the injections under a dissection microscope. After injection, males were placed in individual cages with ample water, food, and cover. No increase in mortality was observed following injection of saline or dsRNA.

### Experimental design

To determine if gene-specific dsRNA could knockdown the abundance of our target protein (EJAC-SP) in the spermatophore, we injected males (from a Texas population of *A. sp. nov.* Tex) with either *ejacsp*-dsRNA or saline (all males were 14 and 22 post-eclosion). Three days post-injection, we began collecting spermatophores from each male (as outlined above). Between days six and eight we allowed both *ejacsp*-dsRNA- and saline-injected males to mate once with a virgin female (as above; all females were 17 to 27 days post-eclosion). Males were frozen at −80C following a successful copulation. Females were given seven days to lay eggs before being scored for possible female sterility (i.e., females were considered sterile if the total number of eggs in her abdomens and those laid was less than 20; most females have >80 eggs). Sterile females were removed from all analyses. Based on the number of eggs laid, we scored females as either having been induced to lay eggs or not. Many times a successful copulation and ejaculate transfer does not result in a female laying any eggs (i.e., a successful copulation with the female having laid few eggs despite many in her abdomen). To remove the effects of these females from our analyses, females had to lay more than 5 eggs per day – which is about the maximum number of unfertilized eggs a virgin female will lay per day.

Protein from each spermatophore for males from each treatment was run on a SDS-PAGE gel and scored for the relative abundance of EJAC-SP (as described above). The relative abundance of EJAC-SP between the saline and *ejacsp*-dsRNA treatments was evaluated with an ANOVA. Also, quantitative real-time PCR (qPCR) was used to evaluate the degree of trypsin-like serine protease knockdown in the accessory gland, testis, digestive tract, and thorax (see below). Finally, we analyzed the relationship between relative expression of EJAC-SP in the ejaculate and the age-specific egg-laying rate for each treatment (i.e., the number of eggs laid per day by females). The difference in egg-laying rates between treatments were analyzed with a one-tailed *t*-test, as we were specifically interested in the question: does knockdown of EJAC-SP protein levels in the male ejaculate result in females laying fewer eggs than expected from matings with saline-injected males? The overall relationship between the relative amount of EJAC-SP and egg-laying rate was assessed with a regression analysis.

### Real-time quantitative PCR (qPCR)

Males were frozen immediately at −80°C after obtaining the final spermatophore. Accessory glands, testes, the digestive tract, and the thorax were dissected from individual males (i.e., the males that induced females to lay eggs and were used in the phenotype analysis) and total RNA was isolated using the RNAqueous 4-PCR kit from Ambion. Kit manual instructions were followed; including the DNase I treatment to remove any DNA from the sample, and final RNA volume was 75 µL. cDNA synthesis was performed on each sample using standard protocols (see above). The concentration of cDNA resulting from these reactions was measured using a NanoDrop ND-1000. Nuclease-free water was added to obtain a concentration of ∼800 ng/µL. qPCR reactions were carried out using a BioRad iCycler iQ multicolor Real-Time PCR detection system using standard protocols, including three technical replicates per reaction.

To determine the correct dilution of cDNA for each tissue type for qPCR a dilution series of 1∶10, 1∶50, and 1∶250 was conducted. The test-gene primers used for this test and the subsequent qPCR were the conserved ovi F 230con and ovi R 500con mentioned above (gene abbreviated as SP in subsequent analyses). These conserved primers were used because they amplify trypsin-like serine proteases in all tissue types, not just the male accessory gland. Moreover, given *ejac-sp* is a male accessory gland biased transcript, primers specific to this transcript would not amplify products in the other tissues, thus eliminating our ability to test if *ejacsp*-dsRNA's effect was specific to the trypsin-like serine proteases in the male accessory gland or all tissues. We found that a dilution of 1∶10 worked for all tissue types except the male accessory gland where a dilution of 1∶250 was used. This was repeated for the control gene, *β-actin* (sense primer, AACTGGGACGACATGGAGAAGAT; anti-sense primer, GCCAAGTCCAGACGC AGGAT), and similar dilutions were used for each gene in each tissue type. Primer efficiencies were acceptable with values between 90 and 103% for both genes in all tissues except for the serine protease primer in the digestive tract (efficiency  = 115.8%).

As for analyses, we conducted qPCR on each of the four tissues from 11 individuals in each of the two treatments (22 individuals in total; saline- and *ejacsp*-dsRNA-injected males at ≥6 days post-injection). A Ct value (i.e., the PCR cycle number where amplification causes the amount of product to cross a set threshold) was calculated for each gene and tissue for each individual (important note: a higher Ct value means a lower amount of gene product in the sample). A ΔCt was calculated as Ct_SP_ – Ct_actin_, resulting in trypsin-like serine protease values being corrected for by the amount of *β-actin* in the sample. Positive values mean the Ct value was higher for the trypsin-like serine protease and thus less transcript than *β-actin* in the sample; the converse is the case if the value was negative. A two-tailed *t*-test was used to compare this metric between the treatments for each tissue type, as any potential bias in protocol or primer efficiencies would be similar within each tissue type. For significant differences, the formula 2^n^, where n is the average number of cycles different between treatments, was used to estimate the fold difference between treatments.
